# Student-Led Fall Prevention Care Management Reduced Fall Risks at Assisted Living Facilities

**DOI:** 10.1093/geroni/igaa057.767

**Published:** 2020-12-16

**Authors:** Hiroko Kiyoshi-Teo, Claire McKinley-Yoder, Erin Lemon, Olivia Ochoa

**Affiliations:** 1 Oregon Health & Science University, Portland, Oregon, United States; 2 University of Portland, Portland, Oregon, United States; 3 Oregon Health Sciences University, Portland, Oregon, United States

## Abstract

Older adults in residential care settings are four times more likely than those not living in care facilities to experience falls. Yet, fall prevention efforts at long-term care settings are under-resourced, under-regulated, and under-studied. To address this gap, we developed and studied the impact of a specialty clinical, Fall Prevention Care Management (FPCM), for nursing students to decrease older adults’ fall risks. We enrolled assisted living residents that facility liaison identified as being high fall risk (fall rates or fall risk were not tracked at the study sites) and MOCA ≥15, in 2 assisted living facilities in Northwest USA. Participants received weekly, 1-hour, individual, semi-structured, Motivational Interviewing-based care management visits by same students over 6 visits. Changes in fall risks were measured by the CDC STEADI assessment (unsteadiness & worry), Falls Self-Efficacy Scale International-Short (FESI-S), and Falls Behavioral Scale (FAB). Twenty-five residents completed the study. Students addressed the following (multiple responses possible): emotional needs (n=23), improved motivation to prevent falls (n=21), and individualized education/coaching (i.e., exercise, mobility aids) (n=10-17). FESI-S score improved from 16.0 to 14.4 (p=.001; decreased fear. FAB score improved from 2.94 to 3.10 (p=.05; more frequent fall prevention behaviors). Frequency of those who felt steady while standing or walking increased (24% to 40%, p=.07) and those who did not worry about falling increased (20% to 36%, p=.08). FPCM clinical offered valuable opportunity to address unmet care needs of older adults to reduce fall risks.

